# Sex Differences in Object Manipulation in Wild Immature Chimpanzees (*Pan troglodytes schweinfurthii*) and Bonobos (*Pan paniscus*): Preparation for Tool Use?

**DOI:** 10.1371/journal.pone.0139909

**Published:** 2015-10-07

**Authors:** Kathelijne Koops, Takeshi Furuichi, Chie Hashimoto, Carel P. van Schaik

**Affiliations:** 1 Anthropological Institute and Museum, University of Zurich, Zürich, Switzerland; 2 Department of Archaeology and Anthropology, Division of Biological Anthropology, University of Cambridge, Cambridge, United Kingdom; 3 Primate Research Institute, Kyoto University, Aichi, Inuyama, Japan; CNR, ITALY

## Abstract

Sex differences in immatures predict behavioural differences in adulthood in many mammal species. Because most studies have focused on sex differences in social interactions, little is known about possible sex differences in ‘preparation’ for adult life with regards to tool use skills. We investigated sex and age differences in object manipulation in immature apes. Chimpanzees use a variety of tools across numerous contexts, whereas bonobos use few tools and none in foraging. In both species, a female bias in adult tool use has been reported. We studied object manipulation in immature chimpanzees at Kalinzu (Uganda) and bonobos at Wamba (Democratic Republic of Congo). We tested predictions of the ‘preparation for tool use’ hypothesis. We confirmed that chimpanzees showed higher rates and more diverse types of object manipulation than bonobos. Against expectation, male chimpanzees showed higher object manipulation rates than females, whereas in bonobos no sex difference was found. However, object manipulation by male chimpanzees was play-dominated, whereas manipulation types of female chimpanzees were more diverse (e.g., bite, break, carry). Manipulation by young immatures of both species was similarly dominated by play, but only in chimpanzees did it become more diverse with age. Moreover, in chimpanzees, object types became more tool-like (i.e., sticks) with age, further suggesting preparation for tool use in adulthood. The male bias in object manipulation in immature chimpanzees, along with the late onset of tool-like object manipulation, indicates that not all (early) object manipulation (i.e., object play) in immatures prepares for subsistence tool use. Instead, given the similarity with gender differences in human children, object play may also function in motor skill practice for male-specific behaviours (e.g., dominance displays). In conclusion, even though immature behaviours almost certainly reflect preparation for adult roles, more detailed future work is needed to disentangle possible functions of object manipulation during development.

## Introduction

Immature individuals prepare for adult life by practising the behavioural skills necessary for survival and reproduction. As a result, sex differences in the behaviour of infants and juveniles often foreshadow sex differences in adult behaviour and social strategies in numerous mammalian species, including primates [1, 2], ungulates [3, 4], canids [5] and delphinids [6, 7]. For example, infant male chimpanzees interact with more social partners, especially adult males, than females, which matches adult sex-specific social tendencies of greater male gregariousness [8]. Similarly, female primates engage more in play parenting (e.g. chimpanzees [9], macaques [10]) and male rhesus monkeys show toy preferences similar to human boys [11]. However, because most studies have focused on sex differences in social interactions (but see [12]), the question is whether or not sex differences in ‘preparation’ for adult life are also present with regards to tool use skills.

In primates, social learning is essential for the transmission and maintenance of tool use in a population [13–17], whereas in tool-using birds, such as woodpecker finches and New Caledonian crows, tool use is acquired developmentally without the need for social inputs [18, 19]. In species with such a strong genetic predisposition, tool use appears early in development [19]. In primates, on the other hand, tool use starts relatively late [20–22], consistent with a cultural interpretation. However, a genetic link can develop gradually through genetic assimilation on top of an existing cultural adaptation (i.e. Baldwin effect [23]). Indeed, recent findings showed a difference in predisposition for tool use between immature chimpanzees and bonobos reflecting adult tool use in these two species [24]. Hence, even socially-learned behaviours can have some genetic basis. This can concern both species differences and sex differences within a species.

Chimpanzees (*Pan troglodytes*) and bonobos (*Pan paniscus*) are our closest living relatives and had a common ancestor about one million years ago [25]. Whereas wild chimpanzees use tools extensively in feeding, social and self-maintenance contexts [26], bonobos use few tools and none in foraging [27]. Moreover, captive bonobos show most tool use in play [28]. Early studies on wild chimpanzees suggested that female chimpanzees are more avid and competent foraging tool users than males: females at Gombe (Tanzania) used tools to fish for termites more often and for longer times than did males [29]. Chimpanzee females spent more time ant-fishing than males at Mahale (Tanzania) [30] and were more efficient at cracking nuts than males at Taï (Ivory Coast) [31]. These findings suggest a sex bias in feeding tool use towards adult female chimpanzees. Tool use in captive bonobos also seems to be female-biased, with females using a greater diversity of tool types than males [28], and females acquiring an experimental tool use task faster and performing more tool use than males [32]. No information exists on sex differences in tool use by wild bonobos [27], but given the rarity of tool use they are unlikely to be pronounced.

In contrast, studies of human children generally report a sex difference in tool use in favour of boys (reviewed in [33, 34]). For example, in a study of three-year-old children, boys were more likely than girls to engage in object-oriented play and to solve a simple problem using a tool [34]. Moreover, girls exposed to abnormally high levels of prenatal androgen engaged in more object-oriented play than unaffected girls [35]. The apparent male-biased sex difference in object manipulation and tool use in human children has been proposed to reflect an ontogenetic adaptation to an adult life in which making and using tools for hunting plays an important role for men, whereas women gather [33]. However, because gathering and especially subsequent processing also are tool-based, alternative hypotheses cannot be excluded.

The goal of this study was to investigate whether sex differences in object manipulation in immature apes function as preparation for tool use in adulthood (‘preparation for tool use’ hypothesis). Moreover, we aimed to shed light on the discrepancy between the reported sex differences in tool use and object manipulation of human children and chimpanzees. Object manipulation (including object play) is considered a precursor to tool use [36–38] and provides a measure of a species’ predisposition to engage with objects [39]. As expected, young chimpanzees show higher rates of object manipulation than age-matched bonobos [24]. This suggests that immature chimpanzees prepare for tool use in adulthood by manipulating objects, whereas bonobos do this to a lesser extent.

To test the ‘preparation for tool use’ hypothesis, information is needed on both sex differences and developmental changes in object manipulation in wild immature apes. We studied object manipulation in immature chimpanzees (*P*. *t*. *schweinfurthii*) at Kalinzu (Uganda) and bonobos at Wamba (Democratic Republic of Congo). Chimpanzees at Kalinzu commonly use tools to harvest army ants [40]. This type of tool use involves the use of a stick or wand of woody vegetation to harvest the aggressive driver ants (*Dorylus* spp.) from their underground nest. Bonobos at Wamba use tools only in non-feeding contexts, i.e. social contexts, comfort and protection from the rain [27].

We tested the predictions of the ‘preparation for tool use’ hypothesis (Table 1a). First, we examined the effects of sex and age on manipulation rates. We predicted a female bias in object manipulation in chimpanzees and no sex difference in bonobos. Moreover, we expected higher object manipulation rates in younger immatures in both species. Second, we investigated species, sex and age differences in manipulation types (i.e. play *vs*. other). We expected more diverse (i.e. less play-dominated) manipulation types in chimpanzees compared to bonobos, as well as more diverse manipulation types in female compared to male chimpanzees. We predicted that, as chimpanzee infants get older, manipulation types would become more diverse and less dominated by play. In bonobos, we expected play to remain the same with age [28]. Third, we assessed species, sex and age differences in object types (i.e. leaf, stick, fruit). We predicted that chimpanzees would interact relatively more with tool-like sticks, whereas bonobos were expected to manipulate all object types. We predicted no sex differences in object types manipulated. Lastly, in chimpanzees, we predicted a developmental change in the object types manipulated, with objects increasingly approximating stick tools used by adult Kalinzu chimpanzees. In bonobos we predicted no effect of age on object types. This study is exploratory in nature, since little is known about the potential role of object manipulation in preparation for tool use. Hence, we measured all types of object manipulation. We aimed to find out whether overall object manipulation, diversity of object manipulation or specific types of manipulation play a role in preparation for tool use.

**Table 1:** a) Predictions of the ‘preparation for tool use’ hypothesis *sensu lato* (F = female, M = male, Young = <3 yrs, Old = >3 yrs), and b) results from this study (✓ = confirmed/significant, ✗ = not confirmed/non-significant).

a) *Predictions*			*Variables measured*	b) *Predictions confirmed*?	
**Species**	Chimpanzee:	Bonobo:		Chimpanzee:	Bonobo:
1. Object manipulation:	>	<	Manipulation rate	✓	✓
2. Manipulation types:	More diverse	More play	Manipulation types	✓	✓
3. Object types:	Mostly sticks	All types	Object types	✗	✓
**Sex**					
1. Object manipulation:	F > M	F = M	Manipulation rate	✗	✓
2. Manipulation types:	F more diverse than M	F = M	Manipulation types	✓	✓
3. Object types:	F = M	F = M	Object types	✓	✓
**Age**					
1. Object manipulation:	Young > Old	Young > Old	Manipulation rate	✓	✓
2. Manipulation types:	More diverse with age	Young = Old	Manipulation types	✓	✓
3. Object types:	More tool-like (sticks) with age	Young = Old	Object types	✓	✓

## Methods

### Ethics Statement

Permission to carry out research at in the Kalinzu Forest Reserve was granted through permits from the National Forestry Authority (NFA) and the Ugandan National Council for Science and Technology (UNCST). Permission to carry out research at Wamba in the Luo Scientific Reserve was granted and approved through permits from the Ministry of Higher Education, University and Scientific Research and the Centre of Research of Ecology and Forestry (CREF).

### Study Areas and Subjects

Kalinzu Forest Reserve is in western Uganda (30° 07’ E, 0° 17’ S [41]). The forest is classified as medium-altitude moist evergreen forest [42, 43]. We collected data on immature members (0.7–7.1 yrs, Table 2) of the main study community (M-group), which consisted of 97 individuals (19 adult males, 29 adult females). Data collection took place from December 2012 to March 2013, covering the late rainy season and dry season (January onwards) [44]. Wamba is in the northern section of the Luo Scientific Reserve in the Democratic Republic of Congo (research camp: 0° 11’ 08” N, 22° 37’ 58” E [45]). The habitat comprises primary, old secondary, young secondary and swamp forest [46]. There are two neighbouring study groups: E1-group (31 individuals: 7 adult males, 9 adult females) and P-group (28 individuals: 5 adult males, 7 adult females). We collected data on immature members (1.2–6.8 yrs, Table 3) of both communities. Data collection took place from May to August 2013, covering parts of the wet and dry seasons (July onwards) [47].

**Table 2:** Information on Kalinzu chimpanzee focal individuals (name, sex, age), name of mother, total observation time per individual, object manipulation rates and mean bout length.

*Name*	*Sex*	*Mother*	*Age (yrs)*	*Observation time (hrs)*	*Manipulation rate (bouts/hr)*	*Mean bout length (min)*
Mugisha	M	Mitsu	0.7	4.7	2.11	3.4
Hayato	M	Haro	0.9	9.7	2.05	1.7
Eta	M	Esunzu	1.3	9.5	0.84	1.4
Picasso	M	Pinka	2.6	9.0	0.89	1.6
Max	M	Mami	3.5	6.0	3.33	2.0
Taro	M	Tae	~5.3	10.0	0.80	1.4
Milk	M	Mitsu	6.1	3.4	0.59	1.4
Ayu	F	Asa	2.6	5.4	0.92	1.0
Iyo	F	Ida	2.9	9.2	0.98	2.7
Gale	F	Gai	4.0	9.7	0.51	2.5
Haruka	F	Haro	4.8	9.4	0.42	2.8
Ua	F	Ume	6.1	5.8	0.35	1.0
Piriko	F	Pinka	~6.8	7.4	0.14	1.0
Iku	F	Ida	7.1	10.0	0.40	1.4
**Mean**			**3.9**	**7.8**	**1.02**	**1.8**

**Table 3:** Information on Wamba bonobo focal individuals (name, sex, age), community (grp.), name of mother, total observation time per individual, object manipulation rates and mean bout length.

*Name*	*Grp*.	*Sex*	*Mother*	*Age (yrs)*	*Observation time (hrs)*	*Manipulation rate (bouts/hr)*	*Mean bout length (min)*
Kale	P	M	Kabo	1.2	12.0	0.50	1.9
Isao	P	M	Ichi	1.3	12.7	0.55	1.9
Seko	E1	M	Sala	1.4	13.2	0.98	3.5
Hideo	P	M	Hide	2.3	12.5	0.64	3.5
Hachiro	E1	M	Hoshi	3.8	13.5	0.37	1.0
Kyota	E1	M	Kiku	3.8	14.5	0.21	1.0
Joe	E1	M	Jacky	6.8	13.5	0.07	1.0
Jolie	E1	F	Jacky	1.4	14.2	0.92	1.2
Fua	E1	F	Fuku	2.4	13.5	0.59	2.0
Otoko	E1	F	Otomi	2.4	13.5	0.89	2.3
Yume	E1	F	Yuke	3.6	13.9	0.94	1.6
Natsuko	E1	F	Nao	4.0	13.6	0.29	2.0
Pipi	P	F	Pao	4.5	12.3	0.16	2.0
Ichiko	P	F	Ichi	4.5	13.2	0.15	2.0
Hideko	P	F	Hide	7.0	12.3	0.16	2.0
Kaboko	P	F	Kabo	7.0	12.2	0.08	1.0
**Mean**				**3.6**	**13.2**	**0.47**	**1.9**

### Data Collection

Behavioural data collection involved focal animal sampling [48]. We collected data on 14 chimpanzees (7 males, 7 females, Table 2) in M-group and 16 bonobos (7 males, 9 females, Table 3) in E1-group (N = 9) and P-group (N = 7). At Kalinzu, we collected 109.3 hrs of observations (mean observation time per individual = 7.9 hr). At Wamba, we collected 210.6 hrs of observations (mean observation time per individual = 13.2 hr). All focal individuals were followed on at least three different days, and observation times were balanced for time of day. Focal individuals were followed for 30–120 min per follow (mean duration: bonobo: 81.7 min, chimpanzee: 75.6 min). Every two min, we recorded the focal animal’s general activity (i.e. feed, rest, move, play, groom) and location (i.e. terrestrial, arboreal). In addition, we recorded all occurrences of object manipulation bouts during focal follows. An object manipulation bout was defined as continuous manipulation of the same (detached) object with hands, feet or mouth. A new bout was scored when the object(s) changed or when there was a break of at least 2 minutes in object manipulation. For all object manipulation bouts, we noted the type of manipulation (i.e. play, explore/touch, tool use, bite/chew, break/pluck, carry, throw/drop) and the type of object (i.e. leaf, stick, fruit, other). *Play*: Manipulating object with no apparent immediate goal, including repetitive movements, alone or together with other individual(s) and often associated with a play face. *Explore/touch*: Non-repetitive touching and exploring of object with hands, feet or mouth without apparent feeding purpose, usually destructive. *Tool use*: Using object as a means to achieve an end (e.g. ant dip, branch drag, penis wipe). *Bite/chew*: Biting or chewing object without ingestion. *Break/pluck*: Breaking (off) object or plucking object without apparent feeding purpose. *Carry*: Carrying object in hands, feet or mouth. *Throw/drop*: Throwing object or intentionally dropping object from an elevated position. We did not include actual feeding behaviour (i.e. ingestion). The category ‘stick’ included woody vegetation with and without leaves. The category ‘other’ included moss, seeds, seedpods and bird’s nest.

### Statistical Analyses

We tested data for normality using a normal probability plot and a Kolmogorov-Smirnov test [49]. We performed statistical tests in IBM SPSS version 21.0. All analyses were two-tailed and significance levels were set at 0.05. We performed a logarithmic transformation for object manipulation rate and all analyses of object manipulation rates were done on the transformed values. We conducted analyses of covariance (ANCOVA) with the fixed factors species and sex, object manipulation rate (bouts/hr) as the dependent variable, and age as covariate. To compare object manipulation rates between the sexes and between age categories (<3 yrs *vs*. >3 yrs) within both species we used Independent Samples T-tests, since inequality of variances and small sample sizes did not allow within species ANCOVAs. The age category cut-off point was chosen at 3 yrs old, since chimpanzees achieve independence in locomotion and nest-building around this age [50, 51]. We excluded one significant outlier individual (i.e. *Max*, Table 2) from the comparison of object manipulation rates between age categories in chimpanzees. This individual was an outlier only in the analysis of age categories. Since exclusion of this individual had no effect on the outcomes of the other analyses of object manipulation rates, we included the individual in these analyses. We correlated object manipulation rates with age using Pearson’s Correlations for both chimpanzees and bonobos.

In addition, we compared object manipulation bout lengths between species, sexes and age categories. We estimated bout lengths based on all occurrences sampling of object manipulation between scans, as well as on the 2-min activity scans. We used the following categories to estimate mean bout lengths: 0–2 min = 1 min, 0–4 min = 2 min, 2–4 min = 3 min, 2–6 min = 4 min, 4–6 min = 5 min, 4–8 min = 6 min, 6–8 min = 7 min, 6–10 min = 8 min, 8–10 min = 9 min, 8–12 min = 10 min. We discarded bouts that either started before the focal follow or were not finished when the focal follow ended. Based on the values for all bouts per individual, we calculated a mean object manipulation bout length for each individual. We performed a logarithmic transformation for mean bout length and all further analyses were done on the transformed values. We conducted analyses of covariance (ANCOVA) with the fixed factors species and sex, object manipulation bout length (min) as the dependent variable, and age as covariate. We correlated mean bout lengths with age using Pearson’s Correlations for both chimpanzees and bonobos.

We compared object manipulation types (i.e. play *vs*. other) between species, sexes and age categories using Chi-squares tests. We also compared manipulation of different object types between species, sexes and age categories using Chi-square tests. We determined the categories in which proportions of object manipulation bouts differed between species, age classes or sexes, by inspecting adjusted residuals (adj. res.), which are approximately normally distributed, and controlled for multiple testing with the improved Bonferroni procedure [52].

## Results

### Object Manipulation

We compared object manipulation rates (Tables 2 and 3) between the two species and sexes controlling for age. In an ANCOVA, object manipulation rates were higher for chimpanzees as compared to bonobos (F [1, 25] = 18.0, *P* < 0.0001) when controlling for age (F [1, 25] = 22.3, *P* < 0.0001), but no sex difference was found (F [1, 25] = 1.3, *P* = 0.259). There was no interaction between Age and Species (F [1, 24] = 0.522, *P* = 0.477) or Age and Sex (F [1, 24] = 0.545, *P* = 0.467). However, there was a significant interaction between Species and Sex (F [1, 25] = 6.0, *P* = 0.021) and we therefore had to compare object manipulation rates in chimpanzees and bonobos separately.

**Fig 1:**
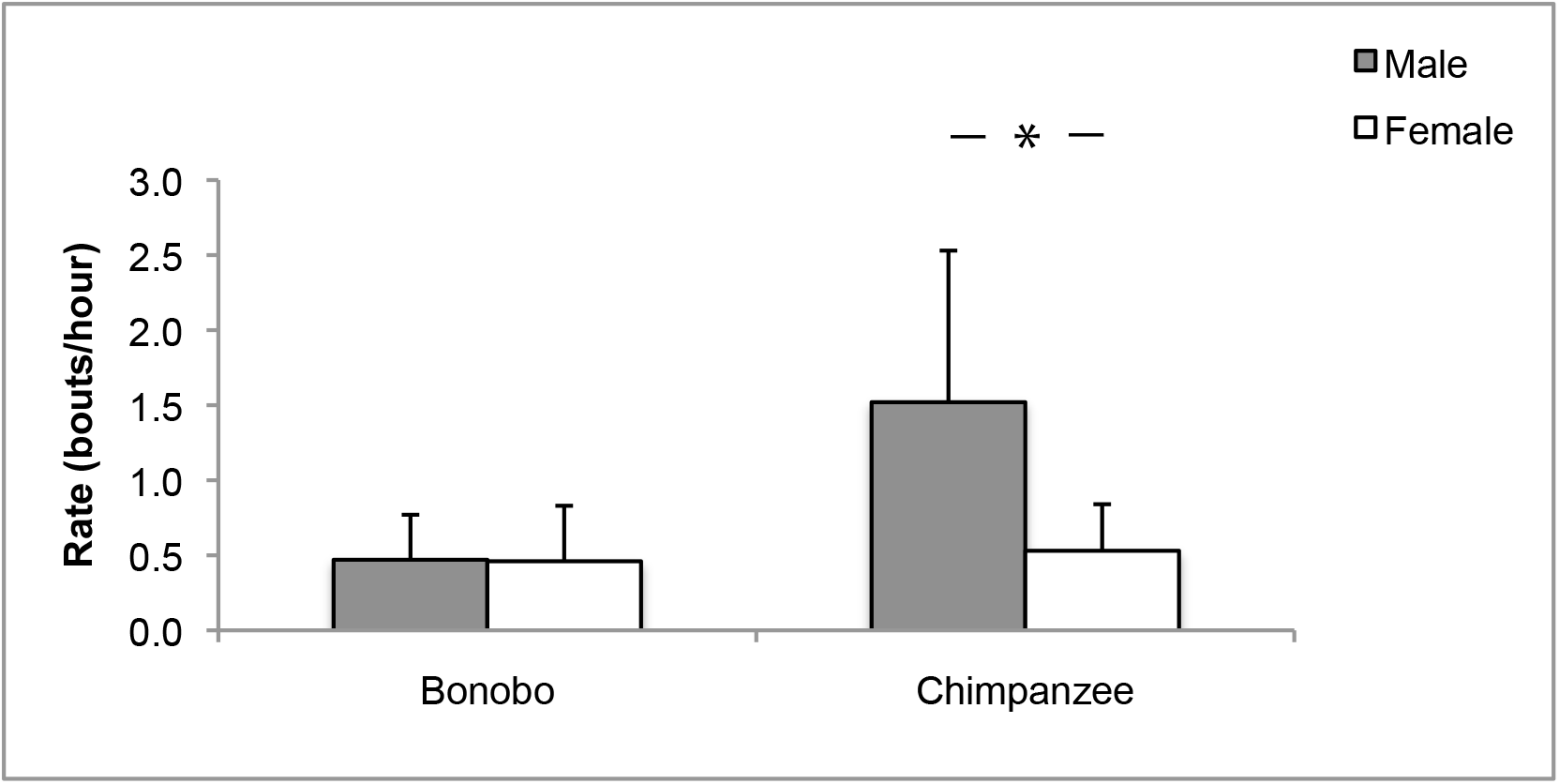
Object manipulation rates in immature male and female chimpanzees and bonobos. Mean object manipulation rates in bouts/hr (SD) for immature bonobos (left) and chimpanzees (right) according to sex (male = grey, female = white). * Independent Samples T-test: *P* < 0.05

When comparing the sexes within species, we found that in chimpanzees mean object manipulation rates were 1.52 ± 1.01 bouts/hr for males and 0.53 ± 0.31 bouts/hr for females. In bonobos, mean object manipulation rates were 0.47 ± 0.30 bouts/hr for males and 0.46 ± 0.37 bouts/hr for females. In chimpanzees, males showed significantly higher object manipulation rates than females (Independent Samples T-test: t = 2.8, df = 12, *P* = 0.019, Fig 1), whereas bonobos showed no significant sex difference (t = 0.15, df = 14, *P* = 0.886, Fig 1).

**Fig 2:**
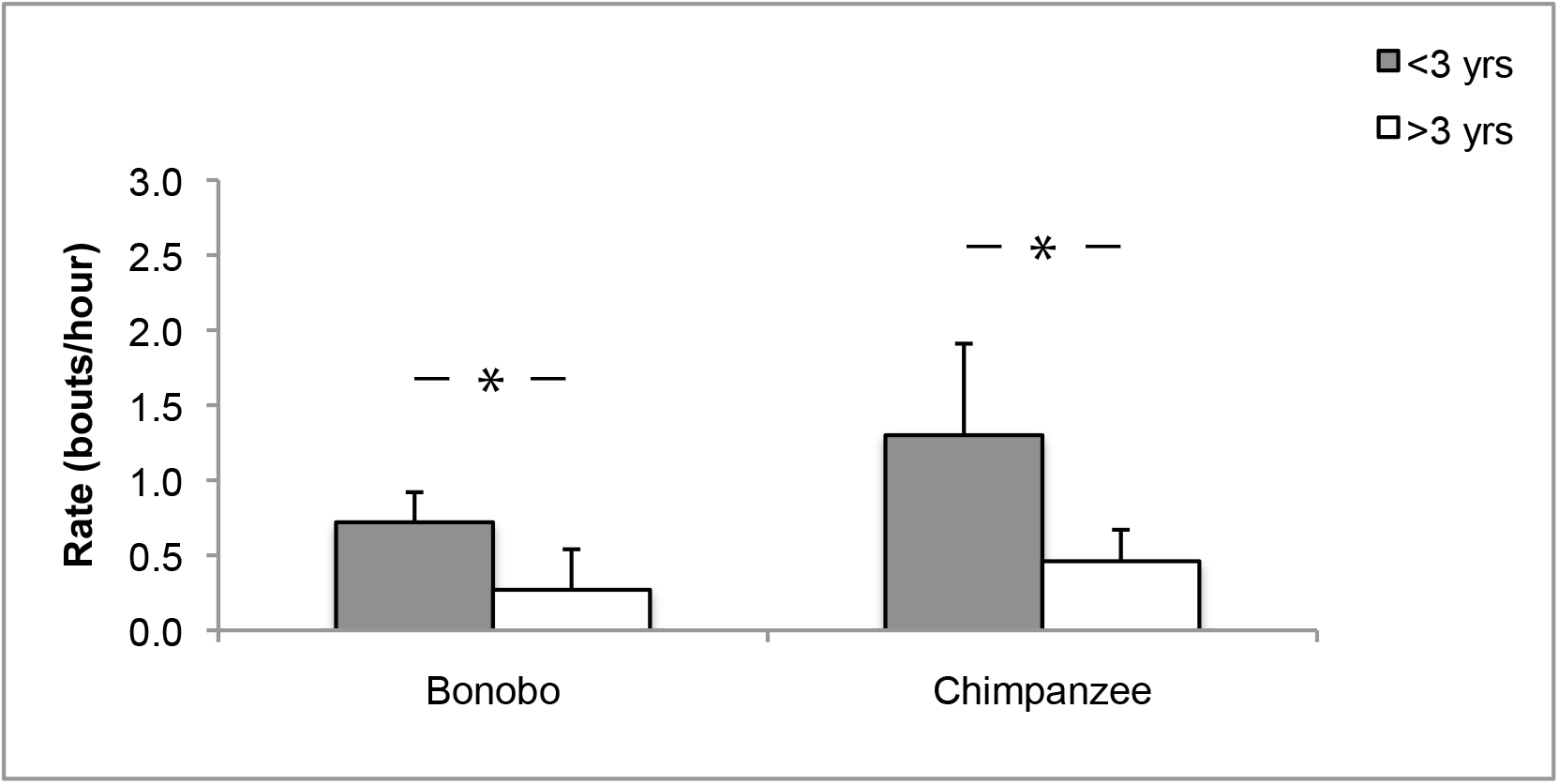
Object manipulation rates in immature chimpanzees and bonobos aged <3 yrs and >3 yrs old. Mean object manipulation rates in bouts/hr (SD) for immature bonobos (left) and chimpanzees (right) in two age classes (<3 yrs old = grey, >3 yrs old = white). * Independent Samples T-test: *P* < 0.05

We examined age differences in object manipulation rates within species. We found a negative correlation between age and object manipulation rate in both bonobos (r = -0.801, N = 16, *P* < 0.0001) and chimpanzees (r = -0.690, N = 14, *P* = 0.006). In chimpanzees, mean object manipulation rates were 1.30 ± 0.61 bouts/hr for immatures <3 yrs old and 0.46 ± 0.21 bouts/hr for immatures >3 yrs old. In bonobos, mean object manipulation rates were 0.72 ± 0.20 bouts/hr for immatures <3 yrs old and 0.27 ± 0.27 bouts/hr for immatures >3 yrs old. Younger immatures showed significantly higher object manipulation rates than older immatures in both chimpanzees (Independent Samples T-test: t = 4.0, df = 11, *P* = 0.002, Fig 2) and bonobos (Independent Samples T-test: t = 4.0, df = 14, *P* = 0.001, Fig 2).

We also investigated object manipulation bout lengths. We found no species difference (F [1, 25] = 0.001, *P* = 0.982, Tables 2 and 3) and no sex difference (F [1, 25] = 0.461, *P* = 0.503, Tables 2 and 3) in mean estimated object manipulation bout length when controlling for age (F [1, 25] = 6.572, *P* = 0.017). There was no interaction between Species and Sex (F [1, 25] = 0.020, *P* = 0.889). Moreover, there was no interaction between Age and Species (F [1, 24] = 0.036, *P* = 0.852) and Age and Sex (F [1, 24] = 1.095, *P* = 0.306). No significant correlations were found between age and bout length in bonobos (r = -0.452, N = 16, *P* = 0.079) or chimpanzees (r = -0.436, N = 14, *P* = 0.119).

### Manipulation Types

**Fig 3:**
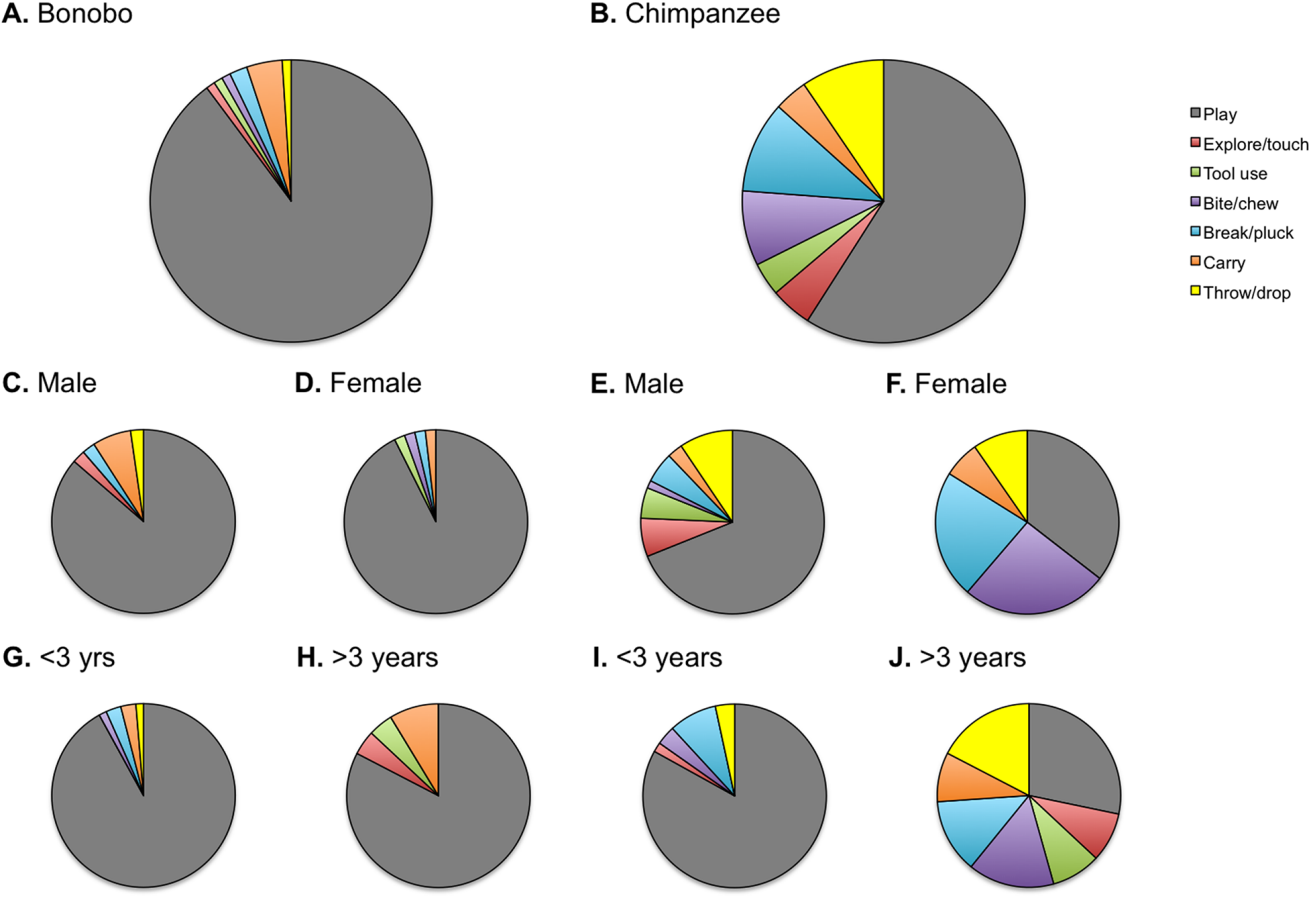
Proportion of object manipulation bouts across manipulation types in immature chimpanzees and bonobos. Object manipulation bouts (%) across object manipulation types for immature bonobos (A) and chimpanzees (B); male and female bonobos (C, D) and chimpanzees (E, F); young (<3 yrs) and old (>3 yrs) immature bonobos (G, H) and chimpanzees (I, J).

We compared object manipulation types between chimpanzees and bonobos. Chimpanzees had larger contributions of manipulation types other than play compared to bonobos (Fig 3A and 3B). We compared the relative contribution of play *vs*. other manipulation types and found that object manipulation by bonobos consisted of more play compared to chimpanzees (χ^2^ = 24.842, df = 1, *P* < 0.0001). In bonobos, males and females did not differ with regards to manipulation types (χ^2^ = 1.027, df = 1, *P* = 0.311, Fig 3C and 3D). In chimpanzees, object manipulation by males contained relatively more play compared to females (χ^2^ = 10.100, df = 1, *P* = 0.001, Fig 3E and 3F). In bonobos, no difference in manipulation types was found between <3 yr vs. >3 yr olds (χ^2^ = 1.694, df = 1, *P* = 0.193, Fig 3G and 3H). In chimpanzees, object manipulation bouts by younger immatures contained relatively more play than those by older immatures (χ^2^ = 32.088, df = 1, *P* < 0.0001, Fig 3I and 3J).

### Object Types

**Fig 4:**
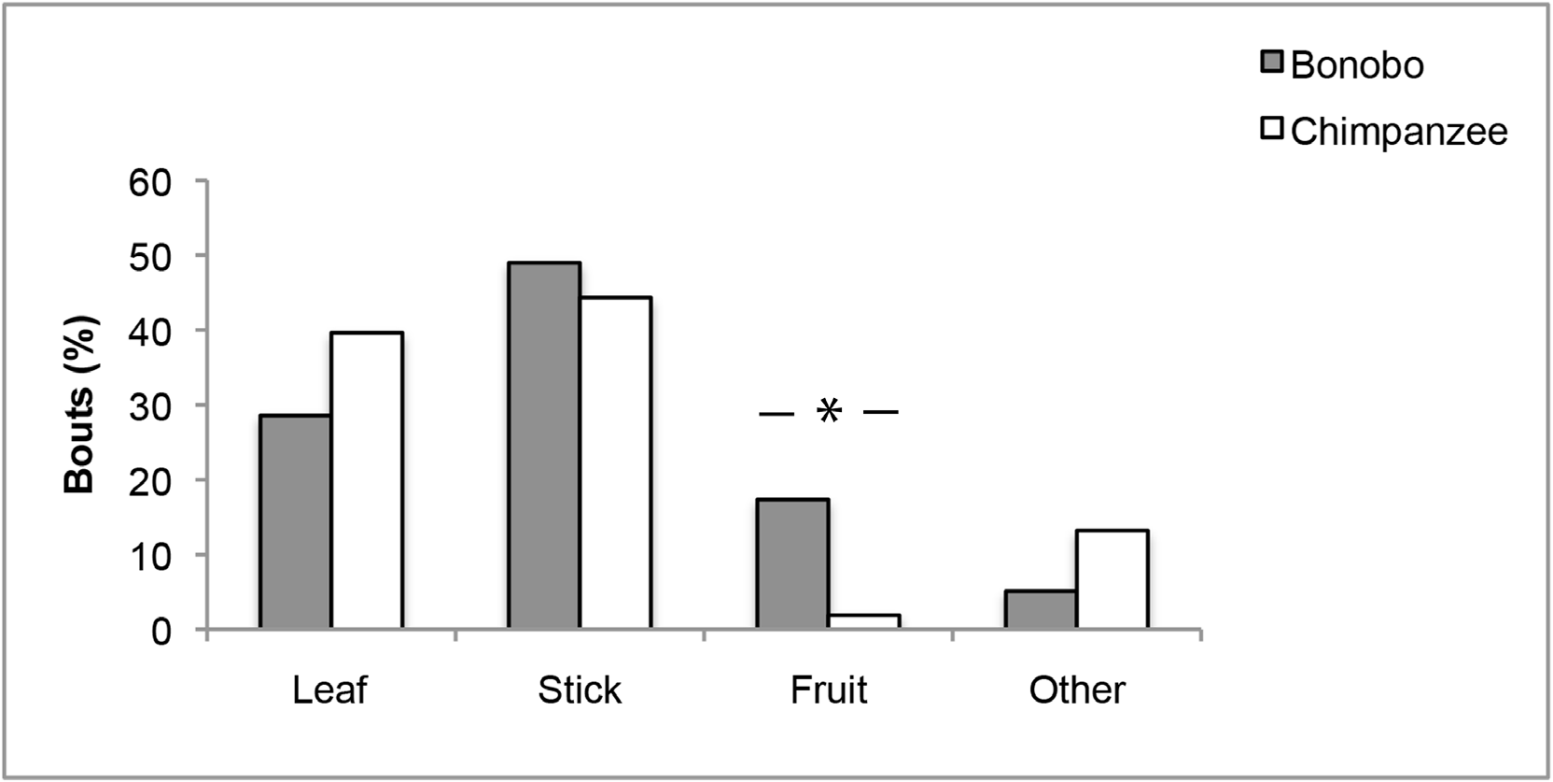
Proportion of object manipulation bouts across object types in immature chimpanzees and bonobos. Object manipulation bouts (%) across object types for immature bonobos (grey) and chimpanzees (white). * Chi-square test: *P* < 0.05

Both chimpanzees and bonobos manipulated leaves, sticks and fruits (Fig 4). The types of objects most often used in object manipulation bouts (chimpanzee: N = 105; bonobo: N = 98) were leaves (chimpanzee: 39.6%; bonobo: 28.6%) and sticks (chimpanzee: 44.3%; bonobo: 49.0%). Bonobos and chimpanzees differed significantly in how much they manipulated different types of objects (χ^2^ = 18.697, df = 3, *P* < 0.0001, Fig 4) with bonobos manipulating relatively more fruits compared to chimpanzees (17.3% vs. 1.9%, adj. res. = 3.8, *P* < 0.0001, Fig 4).

We also compared object types manipulated by males versus females in both species. We found no sex difference in the types of objects manipulated by immature chimpanzees (χ^2^ = 1.1, df = 2, *P* = 0.565) or bonobos (χ^2^ = 3.1, df = 2, *P* = 0.214).

**Fig 5:**
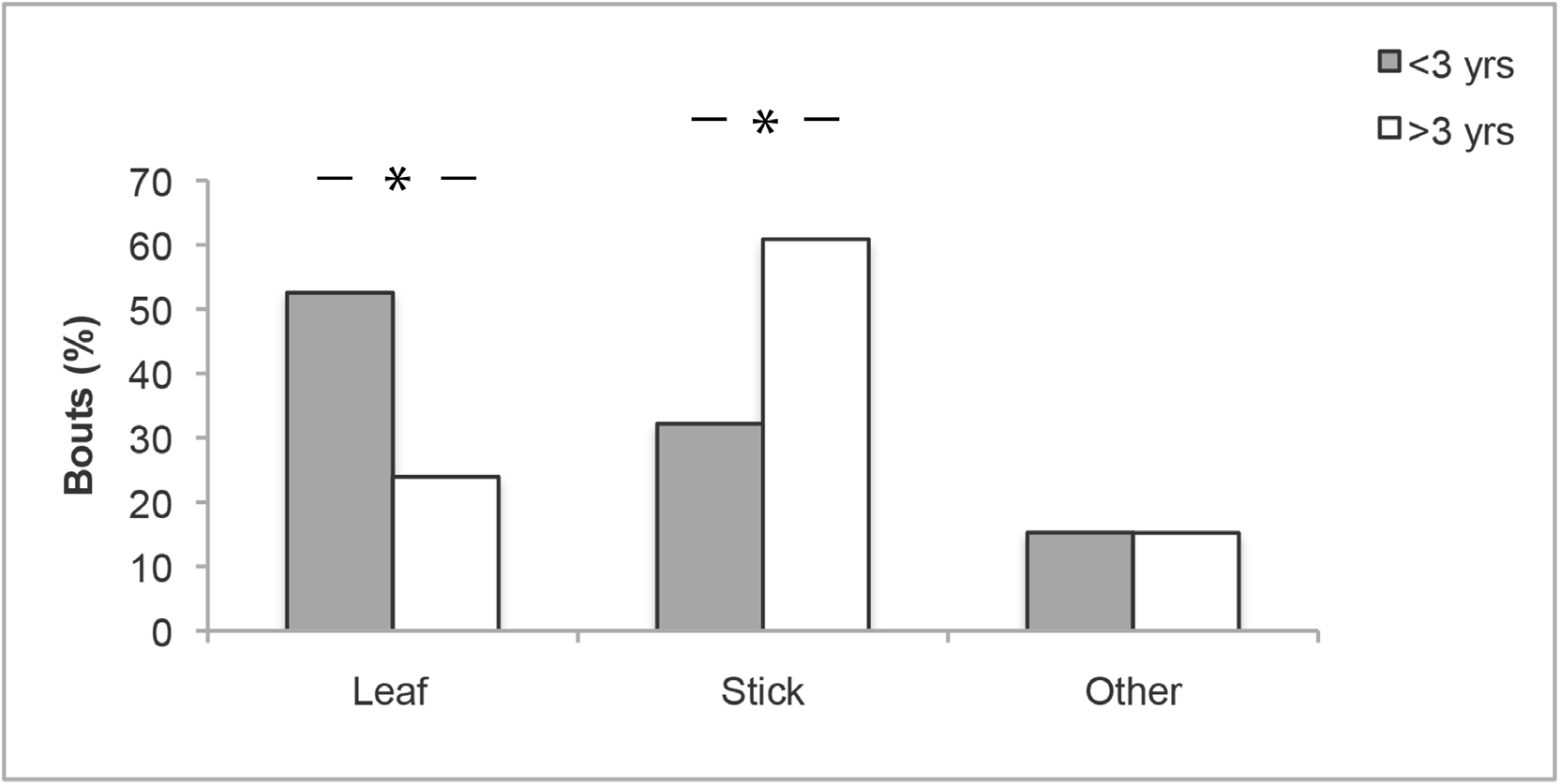
Proportion of object manipulation bouts across object types for chimpanzees <3 yrs and >3 yrs old. Object manipulation bouts (%) across object types for immature chimpanzees <3 yr old (grey) and >3 yr old (white). * Chi-square test: *P* < 0.05

Finally, we examined age effects by comparing types of objects manipulated by individuals <3 yrs compared to >3 yrs old in both species. In chimpanzees, there was a significant age difference in the types of objects manipulated (χ^2^ = 10.0, df = 2, *P* = 0.007, Fig 5). Below the age of 3 yrs chimpanzees manipulated relatively more leaves (adj. res. = 3.0, *P* = 0.003), whereas above 3 yrs they manipulated relatively more sticks (adj. res. = 2.9, *P* = 0.004). In bonobos, no age difference in types of objects manipulated was found (χ^2^ = 3.3, df = 2, *P* = 0.193).

## Discussion

### Object Manipulation

We confirmed most predictions of the ‘preparation for tool use’ hypothesis (Table 1a and 1b). However, surprisingly, we found higher rates of object manipulation in immature male versus female chimpanzees. This is contrary to what we would expect based on the reported sex differences in tool use in adult chimpanzees, but agrees with the sex bias found in human children. What can explain these higher rates of object manipulation in immature male chimpanzees?

Most chimpanzee field studies that addressed the question report a female bias in tool use for foraging [29–31]. Tool use has been hypothesized to provide chimpanzee females with an alternative source of animal protein and micronutrients, which males obtain by hunting [29]. This suggests that Kalinzu females may also be more engaged in tool supported ant-dipping, but no information is currently available on adult sex differences, nor on whether males share meat with females at Kalinzu, like they do for example at Taï [53]. On the other hand, ant-dipping frequencies at Gombe and Bossou (Guinea) showed no significant sex differences [16, 29]. And female chimpanzees at Fongoli (Senegal) hunt more with tools than males [54, 55]. However, given the strong female tendency toward higher rates of insectivory in chimpanzees [29, 30], it is most parsimonious to assume a female bias in adult tool use exists at Kalinzu.

The observed patterns of object manipulation rates among young immature chimpanzees did not seem to reflect preparation for adult subsistence tool use. First, we observed higher object manipulation rates in male as compared to female chimpanzees. Second, we observed similar rates of object manipulation in chimpanzee females and bonobos of both sexes. Third, tool-use related object manipulation first emerged only in older immature chimpanzees. In fact, we recorded only two instances of subsistence-related object manipulation (i.e. ant dip, explorative probe), both involving older immature males aged 5.3 yrs and 6.1 yrs. The latter point suggests that investigation of manipulation *types*, rather than overall manipulation rates, may be more informative in assessing preparation for tool use in immature apes.

### Manipulation Types

The two species differed in their so-called manipulation ‘profiles’ (Table 1b). Object manipulation by bonobos was 90% play compared to 59% in chimpanzees. In bonobos, play-dominated object manipulation was consistent across sexes and ages. In chimpanzees, on the other hand, play accounted for most object manipulation in males (69%), but not in females (36%). Female chimpanzees invested more time in manipulation of objects outside a play context, such as touch/explore, break/pluck and bite/chew. Hence, the more diverse, less play-dominated, manipulation types favoured by immature female chimpanzees compared to males are in line with preparation for tool use in adult life. Due to sample size limitations across the different object manipulation types, these results should be considered preliminary.

The observed male-bias in object manipulation rates in Kalinzu chimpanzees suggests that object manipulation by immature males, especially in play, may reflect practice of general motor skills in relation to male-specific behaviours involving objects, such as dominance displays and predator mobbing [56, 57]. The fact that most object manipulation by males, and by younger immatures in general, was object play, suggests that at least the younger individuals (i.e. who have not yet acquired independence in locomotion and social interactions) primarily practiced general motor and social skills, rather than specific tool use related skills.

Immature females, on the other hand, showed less object manipulation, especially in play, likely due to more diverse and foraging-related activities overall. At Gombe, immature female chimpanzees paid more attention to their mothers using tools in termite-fishing and became proficient tool users at an earlier age than males [12, 58]. Immature females seemed to focus their attention on relevant tool use tasks and thus learned quicker, whereas males seemed to do more undirected exploration in play. It has to be noted that due to the imbalance in age distributions of males and females in our study, more data are needed to confirm the observed sex differences in object manipulation rates and manipulation types.

In addition, we found an effect of age on manipulation types in chimpanzees, but not in bonobos. Object manipulation by older immature chimpanzees ceased to be dominated by play, but contained various other types of manipulation (including tool use). In bonobos, play dominated object manipulation in both younger and older immatures. This result fits with previously reported findings on developmental differences between chimpanzees and bonobos that promote juvenile behaviours and cognitive mechanisms in bonobos throughout life, such as high levels of adult play [59, 60].

### Object Types

Chimpanzee and bonobo immatures engaged with a similar range of objects, but bonobo object manipulation involved relatively more fruits, or fruit parts. Bonobos played with the fruits of 10 tree species versus only two species for chimpanzees. However, a direct comparison is problematic, since fruit availability varies between the two study sites, as well as seasonally. Future research will need to address this in more detail.

Young chimpanzees manipulated and played more with leaves, whereas older immatures engaged more with sticks (Table 1b). In bonobos, no age difference emerged in the types of objects manipulated. Chimpanzees at Kalinzu use foraging tools primarily in ant-dipping. Hence, the main tool type used by adult chimpanzees at Kalinzu is a stick. The increased use of sticks in object manipulation by older immatures suggests that social learning during ontogeny shapes the types of objects young apes interact with, and directs their attention towards relevant objects. Due to the limited number of observations for the different object types these findings have to be considered preliminary.

One promising direction for future research is to compare object manipulation rates and object types manipulated in immatures across chimpanzee study sites with different types of tool use repertoires. At some sites, for instance, adults use mainly leaf-based tools, whereas at others, they rely largely on stick-based tools. Likewise, pounding tool use is common at some sites, but absent at others. If age changes in object manipulation among immatures mimic adult tool use behaviours, we expect young chimpanzees at a site where adult chimpanzees use leaves, but not sticks as tools, to continue manipulating leaves instead of switching to sticks.

## Conclusions

Chimpanzees showed a sex difference in object manipulation, whereas no such difference was found in bonobos. Male chimpanzees showed higher rates of object manipulation than females, which is contrary to expectation based on the ‘preparation for tool use’ hypothesis. However, female chimpanzees showed more diverse types of object manipulation compared to play-dominated object manipulation by males. We propose that object play in younger immatures, especially males, mainly concerns preparation for adulthood in terms of practising general motor skills. Older immature chimpanzees seem to prepare for adulthood by performing more subsistence-related object manipulation. The switch from non-tool to tool materials in immature Kalinzu chimpanzees further suggests that older immatures become more focussed on functionally relevant materials in object manipulation.

The apparent similarity between humans and chimpanzees in the observed male bias in object manipulation is note-worthy. In both species males engage in aggressive dominance displays [61, 62], which sometimes involve the aimed throwing of objects [56, 63]. Similar behaviours are shown in the predator-mobbing context, again predominantly by males [64, 65]. These patterns are consistent with the hypothesis that the motivation of young chimpanzee and human males to throw and hit with objects in play may reflect a preparation for object use in display and defence, which in humans acted as a pre-adaptation to weapon use.

Object manipulation in our closest living relatives may inform us about the possible biological basis of sex differences in object manipulation and tool use in human children. Our finding that in chimpanzee infants, like humans, object-oriented play is biased towards males may reflect a shared evolutionary history for this trait dating back to the last common ancestor of humans and *Pan*. Thus, ‘preparation’ in immatures at different points in their development may be for various different sex-biased adult activities. Resolving this issue in our closest living relatives may help us to identify the functions of the highly debated gender differences among children.

## Supporting Information

S1 Table Object manipulation bouts according to object manipulation type for bonobos. (DOCX)

S2 Table Object manipulation bouts according to object manipulation type for chimpanzees. (DOCX)

S3 Table Object manipulation bouts according to object type for bonobos. (DOCX)

S4 Table Object manipulation bouts according to object type for chimpanzees. (DOCX)
