# Supplementary material for: Sex Differences in Object Manipulation in Wild Immature Chimpanzees (Pan troglodytes schweinfurthii) and Bonobos (Pan paniscus): Preparation for Tool Use?
Source: PLoS One. 2015 Oct 7;10(10):e0139909. doi: 10.1371/journal.pone.0139909 (PMC4596577; doi:10.1371/journal.pone.0139909)
Supplement: S1 Table — (DOCX) [file pone.0139909.s001.docx]

**Table S1. Object manipulation bouts according to object manipulation type for bonobos.** Number of manipulation bouts for different object manipulation types (I: Play, II: Explore/touch, III: Tool use, IV: Bite/chew, V: Break/pluck, VI: Carry, VII: Throw/drop

| *Name* | *Grp.* | *Sex* | *Mother* | *Age (yrs)* | *I* | *II* | *III* | *IV* | *V* | *VI* | *VII* |
| --- | --- | --- | --- | --- | --- | --- | --- | --- | --- | --- | --- |
| Kale | P | M | Kabo | 1.2 | 7 | 0 | 0 | 0 | 1 | 0 | 0 |
| Isao | P | M | Ichi | 1.3 | 6 | 0 | 0 | 0 | 0 | 0 | 1 |
| Seko | E1 | M | Sala | 1.4 | 11 | 0 | 0 | 0 | 0 | 2 | 0 |
| Hideo | P | M | Hide | 2.3 | 8 | 0 | 0 | 0 | 0 | 0 | 0 |
| Hachiro | E1 | M | Hoshi | 3.8 | 3 | 0 | 0 | 0 | 0 | 1 | 0 |
| Kyota | E1 | M | Kiku | 3.8 | 2 | 1 | 0 | 0 | 0 | 0 | 0 |
| Joe | E1 | M | Jacky | 6.8 | 1 | 0 | 0 | 0 | 0 | 0 | 0 |
| Jolie | E1 | F | Jacky | 1.4 | 13 | 0 | 0 | 0 | 0 | 0 | 0 |
| Fua | E1 | F | Fuku | 2.4 | 7 | 0 | 0 | 1 | 0 | 0 | 0 |
| Otoko | E1 | F | Otomi | 2.4 | 11 | 0 | 0 | 0 | 0 | 0 | 0 |
| Yume | E1 | F | Yuke | 3.6 | 11 | 0 | 0 | 0 | 0 | 1 | 0 |
| Natsuko | E1 | F | Nao | 4.0 | 2 | 0 | 1 | 0 | 0 | 0 | 0 |
| Pipi | P | F | Pao | 4.5 | 2 | 0 | 0 | 0 | 0 | 0 | 0 |
| Ichiko | P | F | Ichi | 4.5 | 1 | 0 | 0 | 0 | 1 | 0 | 0 |
| Hideko | P | F | Hide | 7.0 | 2 | 0 | 0 | 0 | 0 | 0 | 0 |
| Kaboko | P | F | Kabo | 7.0 | 1 | 0 | 0 | 0 | 0 | 0 | 0 |
| **Total** |  |  |  |  | **88** | **1** | **1** | **1** | **2** | **4** | **1** |
