# Supplementary material for: Sex Differences in Object Manipulation in Wild Immature Chimpanzees (Pan troglodytes schweinfurthii) and Bonobos (Pan paniscus): Preparation for Tool Use?
Source: PLoS One. 2015 Oct 7;10(10):e0139909. doi: 10.1371/journal.pone.0139909 (PMC4596577; doi:10.1371/journal.pone.0139909)
Supplement: S2 Table — (DOCX) [file pone.0139909.s002.docx]

**Table S2. Object manipulation bouts according to object manipulation type for chimpanzees.** Number of manipulation bouts for different object manipulation types (I: Play, II: Explore/touch, III: Tool use, IV: Bite/chew, V: Break/pluck, VI: Carry, VII: Throw/drop)

| *Name* | *Sex* | *Mother* | *Age (yrs)* | *I* | *II* | *III* | *IV* | *V* | *VI* | *VII* |
| --- | --- | --- | --- | --- | --- | --- | --- | --- | --- | --- |
| Mugisha | M | Mitsu | 0.7 | 10 | 0 | 0 | 0 | 0 | 0 | 0 |
| Hayato | M | Haro | 0.9 | 15 | 0 | 0 | 0 | 3 | 0 | 2 |
| Eta | M | Esunzu | 1.3 | 7 | 0 | 0 | 0 | 0 | 0 | 0 |
| Picasso | M | Pinka | 2.6 | 7 | 1 | 0 | 0 | 0 | 0 | 0 |
| Max | M | Mami | 3.5 | 10 | 2 | 0 | 0 | 1 | 2 | 4 |
| Taro | M | Tae | ~5.3 | 2 | 2 | 3 | 0 | 0 | 0 | 0 |
| Milk | M | Mitsu | 6.1 | 0 | 0 | 1 | 1 | 0 | 0 | 1 |
| Ayu | F | Asa | 2.6 | 2 | 0 | 0 | 1 | 2 | 0 | 0 |
| Iyo | F | Ida | 2.9 | 8 | 0 | 0 | 1 | 0 | 0 | 0 |
| Gale | F | Gai | 4.0 | 0 | 0 | 0 | 2 | 0 | 1 | 1 |
| Haruka | F | Haro | 4.8 | 1 | 0 | 0 | 2 | 2 | 0 | 0 |
| Ua | F | Ume | 6.1 | 0 | 0 | 0 | 0 | 1 | 0 | 1 |
| Piriko | F | Pinka | ~6.8 | 0 | 0 | 0 | 1 | 0 | 0 | 0 |
| Iku | F | Ida | 7.1 | 0 | 0 | 0 | 1 | 2 | 1 | 1 |
| **Total** |  |  |  | **62** | **5** | **4** | **9** | **11** | **4** | **10** |
