# Supplementary material for: Sex Differences in Object Manipulation in Wild Immature Chimpanzees (Pan troglodytes schweinfurthii) and Bonobos (Pan paniscus): Preparation for Tool Use?
Source: PLoS One. 2015 Oct 7;10(10):e0139909. doi: 10.1371/journal.pone.0139909 (PMC4596577; doi:10.1371/journal.pone.0139909)
Supplement: S4 Table — (DOCX) [file pone.0139909.s004.docx]

**Table S4. Individual object manipulation bouts according to object type for chimpanzees.** Number of manipulation bouts for the different object types

| *Name* | *Sex* | *Mother* | *Age (yrs)* | *Leaf* | *Stick* | *Fruit* | *Other* |
| --- | --- | --- | --- | --- | --- | --- | --- |
| Mugisha | M | Mitsu | 0.7 | 7 | 3 | 0 | 0 |
| Hayato | M | Haro | 0.9 | 11 | 3 | 1 | 5 |
| Eta | M | Esunzu | 1.3 | 3 | 3 | 0 | 1 |
| Picasso | M | Pinka | 2.6 | 2 | 6 | 0 | 0 |
| Max | M | Mami | 3.5 | 5 | 12 | 0 | 0 |
| Taro | M | Tae | ~5.3 | 4 | 2 | 0 | 2 |
| Milk | M | Mitsu | 6.1 | 0 | 2 | 0 | 0 |
| Ayu | F | Asa | 2.6 | 2 | 2 | 0 | 1 |
| Iyo | F | Ida | 2.9 | 5 | 3 | 0 | 1 |
| Gale | F | Gai | 4.0 | 1 | 3 | 0 | 2 |
| Haruka | F | Haro | 4.8 | 1 | 3 | 1 | 0 |
| Ua | F | Ume | 6.1 | 0 | 2 | 0 | 1 |
| Piriko | F | Pinka | ~6.8 | 0 | 2 | 0 | 0 |
| Iku | F | Ida | 7.1 | 0 | 4 | 0 | 1 |
| **Total** |  |  |  | **41** | **48** | **2** | **14** |
